# Books and Magazines

**Published:** 1912-02

**Authors:** 


### Books and Magazines.

BRYCE'S POCKET PRACTICE AND VISITING LIST.—C. A. Bryce, A. M., M. D., editor and publisher of *Southern Clinic,* Richmond, Va., price $1, or $1.50 with one year's subscription to *Southern Clinic.* Compiled and arranged with the assistance of Wm. F. Waugh, M. D.; W. T. Marrs, M. D., and Geo. M. Gould, M. D.

This little book is designed to be carried in the physician's breast pocket to insure its full measure of usefulness where memory fails and acute diseases and emergencies allow no time for going back to the office and reading up. Being combined with the Visiting List—a thing that every doctor carries with him—it may be consulted in the patient's presence without arousing suspicion that his doctor is reading up on his case. While certain of the articles have been written by Dr. Bryce and all have been subjected to editorial supervision, the bulk of the book has been written by the three eminent medical men whose names appear on the title page, and which stamp the work as authoritative and representative of the best thought and most advanced views of the ablest men of today.

Dr. Marrs of Peoria, Ill., is an author and writer on medical subjects so well known that he needs no introduction to the medical men who keep in touch with progress.

Dr. Waugh of Chicago is the Dean and Professor of Therapeutics in Bennett Medical College, and as author, editor and teacher has no superior in this country.

Dr. Gould of Ithaca, N. Y., formerly of Philadelphia, who contributes the classic article, "Ophthalmic Emergencies in General Practice," is probably the best known specialist in America. His long service as editor of the *Philadelphia Medical Journal,* and later of *American Medicine,* and as author of Gould's Medical Dictionary, has long ago made him famous in this country and abroad.

With this introduction we present this little book to the profession with a full knowledge that it has been honestly prepared by able men for the help it will furnish their professional brothers in time of need.

---

THREE PLAYS.—By Brieux, Member of the French Academy. With preface by Bernard Shaw. Translated into English by Mrs. Bernard Shaw, St. John Hankin and John Pollock. Brentanos, Publishers, New York, 1912. Price net $1.50.

"In that kind of comedy," writes Bernard Shaw, "which is so true to life that we have to call it tragi-comedy, and which is not only an entertainment but a history and a criticism of contemporary morals, Brieux is incomparably the greatest writer France has produced since Moliere."

The three plays in this volume are a first installment into English of the work of a man who has been admitted into the French Academy for his splendid achievements, and who is recognized by the best thinkers in Europe as one of the profoundest moral forces expressing itself as literature today.

No earnest man or woman can read these plays without being deeply moved and deeply touched. One of the plays was read by Brieux himself, at the special invitation of the pastor, from the pulpit of a church in Geneva. (See editorial in this issue.)

---

SERUM DIAGNOSIS OF SYPHILIS AND BUTYRIC ACID TEST FOR SYPHILIS.—By Hideyo Noguchi, M. D., M. Sc., Associate Member of The Rockefeller Institute for Medical Research, New York. Fourteen illustrations. J. B. Lippincott Company, Philadelphia and London, 1912. Price $2.50. Dedicated to Dr. Simon Flexner, by his pupil.

The object of this book is, first, to give a brief yet adequate account of the principles of serum hæmolysis and of the behaviors of the combinations of antigens and antibodies toward hæmolysis, so essential for a proper understanding of the subject, discussing at some length the quantitative relationship of the factors playing a part in these phenomena, an aspect of the subject that has not perhaps received the consideration that it deserves; and, secondly, to give in detail the technic of Wassermann's method and of the method recommended by the author.

The author has endeavored to treat the subjects of this book in such a manner as to make it suitable for the use of practicing physicians and students, and at the same time with sufficient comprehensiveness to render it useful to laboratory workers.

The last chapter is a digression from the main subject, being devoted to a description of a chemical test for syphilis. The application of this test in the examination of cerebro-spinal fluids is very simple and numerous trials have shown it to be an actual aid in the diagnosis of parasyphilitic affections of the central nervous system.

In the appendix the author has given an extensive bibliography, which should be of value to readers desiring further knowledge on the subject of the complement-fixation test for syphilis and certain closely allied problems of immunity.

---

CASE HISTORIES IN MEDICINE.—Illustrating the diagnosis, prognosis and treatment of disease. By Richard C. Cabot, M. D., Assistant Professor of Clinical Medicine, Harvard Medical School. Second edition revised and enlarged. Boston, 1911. W. M. Leonard, Publisher. Cloth, 295 pages. Price $3.

This volume is the fourth of the Case History Series. One hundred case histories have been selected to cover well the field of general medicine. These have been classified in nine chapters under the headings, Infectious Diseases, Diseases of Gastro-Intestinal and Biliary Tract, Diseases of the Urinary Tract, Diseases of the Circulation, The Respiratory System, The Nervous System, etc. A final chapter, Notes on Drug Therapy, is of marked interest.

Dr. Cabot states that he has prepared this book with practitioners primarily in mind, and that he has gone into details of prognosis and treatment, "what the patient and his family want, more thoroughly."

The author's experience as a teacher of post-graduate as well as undergraduate classes and his well known success in the use of the Case History method lead us to expect what we actually find in the book, an interesting and valuable post-graduate course in general medicine.

